# Respiratory Status in Children and Exposure to Animal Allergens—The Problem of Reverse Causality in Cross-Sectional Studies

**DOI:** 10.3390/children11080941

**Published:** 2024-08-05

**Authors:** Agata Wypych-Ślusarska, Karolina Krupa-Kotara, Klaudia Oleksiuk, Joanna Głogowska-Ligus, Jerzy Słowiński

**Affiliations:** Department of Epidemiology, Faculty of Public Health in Bytom, Medical University of Silesia, 40-055 Katowice, Poland; awypych@sum.edu.pl (A.W.-Ś.); koleksiuk@sum.edu.pl (K.O.); jglogowska@sum.edu.pl (J.G.-L.); jslowinski@sum.edu.pl (J.S.)

**Keywords:** asthma, bronchitis, chronic cough, cross-sectional study, reverse casuality

## Abstract

Background: Some epidemiological studies suggest that early exposure to animal allergens during infancy reduces the risk of bronchial asthma in school-age children. However, the observed associations in some cases may be an effect of the study used (epidemiological observational studies, especially a cross-sectional study) and indicate reverse causality. Aim: This study aimed to determine the association between exposure to animal allergens and the prevalence of respiratory diseases, including bronchial asthma, considering the potential impact of reverse causality on the observed relationships. Material and methods: An analysis of data from a cross-sectional epidemiological study conducted in 2020 involving 3237 primary school students aged 7–15 years in the Silesian Province (Southern Poland) was carried out. The parents of students completed a questionnaire based on The International Study on Asthma and Allergies in Childhood (ISAAC). The relationship between the occurrence of chronic cough, wheezing, and dyspnea in the last 12 months, night waking due to dyspnea, and asthma in the presence of pets was assessed. Exposure to animal allergens was determined by answering the question, “Are there any furry or feathered animals in the home?” with three response options: “yes; they have been in the past; no” (Scenario 1). For the analyses and to reveal a potential reverse causality effect, the last two response categories regarding pet ownership were combined to form a “no” category in Scenario 2, and the first two answers were combined into a “yes” category in Scenario 3. A chi-square test was used to assess the relationship between variables, and a statistical significance level of *p* < 0.05 was adopted. Results: Chronic cough affected 9.5% of children, wheezing in the last 12 months—9.2%, night waking due to dyspnea—5.8%, dyspnea in the last 12 months—4.8%, bronchial asthma—9.2%. Analysis considering the category of having or not having pets (yes vs. no) showed that bronchial asthma was statistically significantly more common in children who did not have pets at home (10.9% vs. 7.9%, *p* = 0.002). A similar situation was observed for wheezing in the past 12 months (10.7% vs. 8.1%; *p* = 0.01) and nocturnal awakening due to dyspnea (6.8% vs. 5.1%, *p* = 0.03). No statistically significant differences were observed for the other symptoms. Analysis by time of pet ownership (a. present; b. present but in the past; c. not present) highlighted similar relationships. Asthma (a. 7.7% vs. b. 13.4% vs. c. 7.7%; *p* = 0.004), wheezing in the past 12 months (a. 8.1% vs. b. 8.9% vs. c. 10.9%, *p* = 0.03) and night waking (a. 5.0% vs. b. 4.5% vs. c. 7.1%; *p* = 0.04) were more common in children without pets and those who had owned pets in the past. The highest proportion of children with asthma was in homes where pets were present in the past. Conclusions: Analyses indicating a relationship between a higher prevalence of asthma and some respiratory symptoms, and the absence of pets cannot be considered as a casual association. The analysis conducted did not reveal a reverse causality effect. The results of observational epidemiological studies, especially a cross-sectional study, should always be interpreted with caution, considering possible distortions and conclusions drawn.

## 1. Introduction

Scientific studies indicate positive health effects of having pets around both children and adults. Owning a cat or dog positively influences physical activity, lowers blood pressure, reduces the risk of obesity, reduces anxiety, and shapes pro-social behavior, reducing the risk of social isolation among the elderly [1,2,3,4]. However, pet ownership is not advisable in every case. Regarding childhood respiratory diseases or the occurrence of allergic diseases, doctors tend to recommend limiting contact due to exposure to animal allergens and possible aggravation of the disease or symptoms [5]. Also, the results of scientific studies do not bring a clear position on this issue due to the often-divergent conclusions of observations and the different methodological scenarios considered [6,7,8].

The multiplicity of the latter deepens the discussion of the impact of exposure to animal allergens on respiratory conditions in children [5,6,7,8]. Research in this area can consider prenatal and postnatal exposure, exposure that only appeared in early childhood, resulting from the presence of pets such as dogs, cats, or rodents, but also from living in agricultural areas and contact with farm animals [9,10]. If other risk factors are added, such as allergic diseases or asthma in the child’s mother, you can obtain several research patterns that often yield contradictory and inconclusive results [9,11]. For example, a study of 7360 children aged 0–8 in China found that early exposure to animal allergens (cat or dog) significantly increased the risk of wheezing, dry cough, and rhinitis and almost quadrupled the risk of asthma [6]. Another study, also conducted in China in a cohort of 1611 school-aged children, makes similar observations, indicating that the presence of pets is associated with an increased risk of respiratory symptoms [7]. In contrast, a study in a cohort of Swedish children indicated a dose-response relationship between more pets and a decrease in allergy, asthma, and respiratory symptoms [8]. In this case, the animals had a protective effect. Finnish researchers came to similar observations, indicating that having a dog or cat in the home reduces the risk of asthma, atopic sensitization, allergic rhinitis, and atopic eczema [12].

Some studies indicate that exposure to animals in early childhood promotes the development of the immune system by increasing the diversity of the gut microbiome [13,14]. This observation has its origins in the so-called hygiene hypothesis, indicating that excessive cleanliness and a sterile environment can lower the threshold for immune adaptation, thereby causing the body to overreact to substances that are not recognizable (pollen, animal allergens) [15].

However, the observed correlations in some cases may be the result of the study model used. One of the more commonly used studies to analyze relationships between different variables is the cross-sectional study. However, it requires knowledge of the advantages and limitations of the research protocol used, as well as the researcher’s awareness that observed relationships between variables may indicate reverse causality [16]. Cross-sectional studies on asthma and respiratory symptoms often rely on standardized questionnaires to collect data on the topic [17,18,19]. An example of such a study is the International Study of Asthma and Allergies in Childhood (ISAAC). At the same time, exposure information from such studies will be limited only to available data. Assessment of exposure and health effects occurs at the same point in time. Long-term follow-up and direct determination of incidence are, therefore, not possible. In addition, exposure information itself often remains limited. The dose of exposure or the exact time of exposure is not always known. Often, the available data are limited to information about the existence or absence of a given exposure [16]. In the case of observations indicating a protective effect of exposure to animal allergens in relation to bronchial asthma, the time of exposure and current pet ownership may be unknown. The question “whether furry or feathered animals are present in the child’s home” does not yield information about the length of potential exposure. Moreover, a negative answer alone does not rule out past pet ownership. This fact can affect the analyzed data and lead to distorted conclusions resulting from reverse causality. If this is the case, cross-sectional studies analyzing the relationship between exposure to animal allergens and the occurrence of asthma, and respiratory symptoms may point to the protective nature of exposure. In effect, it may be due to limiting or depriving children of contact with animals at the time of the disease diagnosis. Thus, a result indicating a lower incidence of asthma in a group of children with animals will not necessarily imply a protective effect of animal allergens, merely an effect of removing exposure in a group of children with more severe cases of the disease.

Given this information, the aim of this study was to evaluate the relationship between exposure to animal allergens and the incidence of respiratory diseases, including bronchial asthma, considering the potential impact of reverse causality on the observed relationships.

## 2. Materials and Methods

### 2.1. Characteristics of the Study Group

We analyzed data from a cross-sectional epidemiological study conducted in 2019 with 3237 elementary school students aged 6–16 years in Silesian Province (Southern Poland). The average age of the subjects was [Mean ± SD: 10.6 ± 2.2 years]. The study group included 1665 girls (51.4%) and 1572 boys (48.6%). Most children resided in cities (91.5%). Data on children’s respiratory symptoms and bronchial asthma, as well as exposure to animal allergens (presence of pets), were collected using a questionnaire that was filled out by the children’s parents.

### 2.2. Eligibility Criteria

This study used group random sampling: first, localities were selected from each county of the Silesian Province, and then an invitation to participate in this study was sent to principals of schools located in the drawn localities. Filling out the online questionnaire by parents was tantamount to giving their consent to participate in this study. Parents were informed prior to the survey about its purpose and how the data would be used. The method of completing the questionnaire was very intuitive and, therefore, did not include instructions for completion, as one selected answer had to be ticked or the data had to be typed in independently.

This study was conducted in accordance with the provisions of the Declaration of Helsinki. In addition, considering the Act of 5 December 1996, on the professions of physician and dentist (Journal of Laws of 2011, No. 277, item 1634, as amended), this study is not a medical experiment and does not require the approval of the Bioethics Committee of the Silesian Medical University in Katowice.

### 2.3. Research Tool

The research tool was an anonymous questionnaire. Questions about respiratory symptoms and asthma were based on the form used in the International Study of Asthma and Allergies in Children (ISAAC) (English, full version of the ISAAC questionnaire available at: https://isaac.auckland.ac.nz/resources/tools.php?menu=tools1 (date of access: 9 July 2024)) [17]. The survey was conducted by CAWI using the available MS 365 EDU Forms software (version 19041.0) tools. Bronchial asthma was defined by a positive response to the question, “Has a doctor ever diagnosed a child with asthma?”. Among the respiratory symptoms analyzed were the occurrence of wheezing and shortness of breath in the past 12 months, nocturnal awakenings due to wheezing in the past 12 months, and chronic cough, which was defined as a cough that lasted at least three months outside of cold periods. The relationship between the occurrence of chronic cough, wheezing in the last 12 months, dyspnea in the last 12 months, night waking due to wheezing, and bronchial asthma in the presence of pets were assessed. Regarding the determination of exposure to animal allergens, three scenarios were adopted to analyze data on the presence of animals. Scenario 1 included 3 responses to the question “Are there any furry or feathered animals in the home: ‘yes; they have been in the past; no’”. Scenario 2 combined the answers “they have been in the past + no” into one category, “no”. Scenario 3 defined exposure through the presence of pets by combining the category “yes + they have been in the past” into a “yes” option. The invited combinations were intended to indicate possible differences in responses depending on the chosen category and to reveal a potential reversal casualty effect.

All data were properly coded, thus preventing the identification of patients in accordance with the Personal Data Protection Act of 29 August 1997 (Journal of Laws 1997, No. 133, item 883).

### 2.4. Statistical Analyses

Statistical calculations were made using STATISTICA 13.3, Stat Soft Poland. The data were presented in numerical-percentage notation—n (%). The prevalence of asthma and respiratory symptoms were compared according to the presence of animals in the homes of the children studied. The chi-square test was used to evaluate the relationship between qualitative variables. The effects of exposure to animal allergens (presence or absence of animals) on the occurrence of respiratory symptoms and asthma in the three scenarios were verified using logistic regression. Adjusted odds ratios (by sex and age) were estimated with 95% confidence intervals (OR, 95%CI). Statistical significance was determined at *p* < 0.05.

## 3. Results

The prevalence of respiratory symptoms and asthma according to gender, place of residence, and age is presented in Table 1. Wheezing, dyspnea in the last 12 months, and asthma occurred statistically significantly more often in the boys’ group than among girls. Chronic cough, wheezing in the last 12 months, and waking up at night due to wheezing were more common among children in the youngest age group.

Table 2 shows data on the presence or absence of pets in children’s apartments according to the early scenarios: Scenario 1 considers three categories of pet ownership: “present”; “present but in the past”; and “no” pets; Scenario 2, in which the categories “present but in the past” and “no” were treated as the absence of pets in children’s apartments; and Scenario 3 considers ever having pets. More than half of the children (55.7%) owned pets at the time of the survey, and for 62%, pets were present sometime in the past.

Due to the observed differences in the prevalence of selected respiratory symptoms and bronchial asthma in boys and girls and by age group, the prevalence of animals was assessed by gender and age (Table 3).

Pets are more often owned by girls than boys; at the same time, in the boys’ group, pets were more often present in the past. At the same time, in the youngest group (6–9 years old), where the prevalence of most of the analyzed respiratory symptoms was the highest, there was a lower percentage of children currently owning pets compared to older age groups. The observed differences proved to be statistically significant.

The incidence of respiratory symptoms and asthma was then analyzed according to the scenario (Table 4).

It has been observed that, regardless of the scenario, children whose homes had or have ever had pets present were less likely to have asthma and less likely to experience wheezing or night waking caused by this. Logistic regression analysis (Table 5) confirmed the importance of pet ownership in lower asthma prevalence, but only for Scenarios 1 and 2, in terms of current ownership versus no pets. For wheezing in the last 12 months, it was also noted that the presence of pets reduced the risk of the prevalence of this symptom, as in waking up at night due to wheezing, but only for Scenarios 1 and 2. At the same time, it was noted that the presence of animals in the past, compared to no animals in Scenario 1, did not affect the incidence of respiratory symptoms and asthma.

## 4. Discussion

The aim of this study was to evaluate the relationship between exposure to animal allergens and the incidence of bronchial asthma and respiratory symptoms and to reveal the potential presence of reverse causality in the analyses performed.

Simple independence analyses revealed no differences between the place of residence (urban–rural) and the incidence of wheezing, dyspnea, chronic cough, or asthma. This is undoubtedly related to the region in which this study was conducted, namely, Upper Silesia. This is an industrialized region, and often, the differences between the nature of rural and urban localities are not as pronounced as in other regions of Poland. However, it was noted that boys were statistically significantly more likely to have asthma, wheezing, and dyspnea in the last 12 months. This observation is consistent with the results of other studies, which show gender-specific differences in the prevalence of wheezing. These are explained by genetic differences and differences due to lung morphology or puberty between boys and girls [18]. Similarly, regarding asthma, the results of epidemiological studies here are consistent: boys and children from younger age groups are more often affected [19,20].

It was also observed that asthma is less common in children who have pets. However, due to the nature of this study (cross-sectional study), this observation cannot be taken as proof of a cause-and-effect relationship. The lower prevalence of asthma in the presence of pets in the study protocol used does not automatically mean that exposure to animal allergens is protective against the disease. The lower prevalence of asthma in the presence of pets in this study should not be interpreted as a cause-and-effect relationship. In a cross-sectional study, such a result does not mean that exposure to animal allergens protects against asthma. The limitations of the cross-sectional study have already been mentioned in the introduction to this paper. However, it is worth reiterating the most important one, which is the lack of temporal sequencing—information on exposure and health effects is measured at a single point in time, as well as limited information on exposure. This juxtaposition means that reverse causality can occur. Regarding analyzing the relationship between exposure to animal allergens and the occurrence of asthma and respiratory symptoms, it is not always possible to be certain about the sequence of events—whether the presence of animals protected against the disease or whether it was the disease and its exacerbations that prompted the children’s parents to limit contact with animals.

In science, the hygiene hypothesis and its continuation in the form of the old friend’s hypothesis are well known. Both try to indicate the importance of increased microbial stimulation in the prevention of allergic diseases and asthma. The hygiene hypothesis was first proposed by Strachan in 1989, and it suggested that infections early in life strengthened the immune system, protecting against the development of allergies [21]. This hypothesis has been confirmed by several epidemiological studies showing that reduced exposure to common bacterial and parasitic pathogens could lead to the development of immune hypersensitivity mechanisms [14,22,23]. The risk of asthma and allergic diseases has, therefore, been attributed to an excessively hygienic environment. A positive effect of farm residence on asthma and allergic diseases was observed, which was explained by the presence of animals and the immunomodulatory properties of the endotoxin they emitted [24]. With the development of civilization, urbanization, and the adoption of the so-called Western lifestyle, one could observe a tendency to stay and live in an increasingly hygienic environment. As a result, the microbial depletion of the environment caused depletion of immune memory and Th1/Th2 lymphocyte balance [24]. The perpetuation of the Th2 lymphocyte phenotype dominant in fetal life can lead to the development of IgE hypersensitivity to common allergens [25]. The old friend’s hypothesis emphasized the importance of proper and undisturbed exposure to microorganisms in the prevention of allergic diseases or asthma [26]. Studies indicate that most microorganisms that modulate the immune system and are components of a healthy microflora come from mothers, families, and the environment, including animals [26,27]. Thus, the hygiene hypothesis and the former pet’s hypothesis have evolved into a microbiotic approach. Many epidemiological studies indicate that the presence of pets can provide immunological benefits [8,12,13,14,27]. However, these are still observations that do not entitle doctors to recommend their patients with allergic diseases or asthma to increase contact with animals.

To verify whether reverse causality was a possible influence in this study, three scenarios were used, considering different options for pet ownership. Scenario 1 was the baseline, unmodified scenario, and it was from this scenario that the original observation indicating a lower prevalence of asthma in the group of children who currently own pets was derived. The second scenario was assumed, referring to the time of this study—whether pets were currently in the child’s residence or not. The third scenario analyzed the situation in which pets were ever present in the past. Surveys most often focus on asking about the current presence of pets or over a recent, short period of time [28,29]. However, theoretically, a question about the presence of pets in any period of a child’s life cannot be excluded in questionnaire studies, especially for case-control studies, so Scenario 3 was additionally proposed. However, none of the scenarios excluded the baseline observation (from Scenario 1), indicating a lower frequency of asthma, wheezing in the last 12 months, and waking up at night due to wheezing in the group of children with pets. This may suggest that the study conducted is free of reverse causality. It is true that some animals were present at some point in the past, but this fact did not affect the results of the observations. It is also unknown what the reason for the current absence of pets was in the case of their previous presence. However, an interesting regularity was noted between the age of children and pet ownership. Children aged 6–9 years were statistically significantly more likely to have symptoms of wheezing and chronic cough than in groups of older children, and these children were also less likely to own pets. This does not automatically imply a causal relationship between these observations, as the cross-sectional study model brings too many unknowns in this regard.

The results of the present study indicate a lower prevalence of asthma, and some respiratory symptoms differ from some observations. In a cross-sectional study of Hungarian children, dog ownership was a risk factor for asthma, while the presence of rodents reduced the risk [30]. In contrast, a study in Italy indicated that exposure to a cat but not a dog increased the risk of asthma and allergic diseases [31]. On the other hand, a study by Svanes et al. analyzing the relationship of asthma incidence to the presence of pets at different periods of life showed that the potential pro-toxigenic effect of animals could be the result of selective avoidance of exposure to animal allergens [32]. It is, therefore, possible that a similar situation also occurred in our own study. Perhaps a prophylactic behavior of avoiding contact with animals was behind the lack of pet ownership in a situation where either parent was allergic or had bronchial asthma. However, the study protocol did not provide such precise questions, so a detailed analysis could be the canvas for future research.

### Strengths and Limitations

A strength of the study conducted is that it draws attention to potential distortions resulting from the study model used. The authors of this paper hope that this will sensitize other researchers to the problem of drawing too hasty conclusions about the existence of a cause-and-effect relationship. In addition, this study included a large group of children, and data on respiratory and asthma symptoms were collected using standardized questions from the ISAAC questionnaire. Three simple scenarios were used to highlight the potential effect of reverse causality.

However, this study has several limitations due to the research model used. The cross-sectional study is one of the simplest epidemiological studies, and its biggest limitation is incomplete information on exposure. In the case of the present study, the time of exposure was not specified, the type of animal owned was not known, nor was the location of the animal (inside or outside the home). It is also unknown when contact with the animal ceased and what caused it. The absence of such questions was since the analysis conducted was part of a larger study, the main objective of which was to determine the socioeconomic determinants of asthma. For the purposes of this study, therefore, the focus was on a certain scope, covering only the available information.

Despite the limitations, a cross-sectional study can provide hypotheses for further analysis of the etiology of diseases and establish causal relationships. Rejection or denial of the validity of the use of a cross-sectional study in assessing relationships between variables and its scientific usefulness may lead to the rejection of evidence that may be helpful in establishing causality [33].

There are also practical implications from this study. Firstly, it is a good training article for young and novice researchers. Secondly, as mentioned earlier, it sensitizes the proper interpretation of the results of a cross-sectional study. Undoubtedly, the study presented here can inspire further research on the relationship between exposure to animal allergens and the occurrence of respiratory diseases and symptoms. It would be good if future projects took into account questions regarding the duration of exposure, the type of animals, the place of residence and its nature (e.g., living on a farm), and potentially other factors such as the genetic background of these diseases, exposure to tobacco smoke, and presence of molds in buildings.

## 5. Conclusions

Asthma, wheezing in the last 12 months, and waking up at night due to wheezing are less common in children with pets. Analyses indicating an association between a higher prevalence of asthma and certain respiratory symptoms, and the absence of pets cannot be treated as a causal association. The analysis conducted did not reveal a reverse causality effect. However, it should be remembered that the results of observational epidemiological studies, especially cross-sectional studies, should always be interpreted considering possible biases and conclusions drawn with caution.

## Figures and Tables

**Table 1:** Prevalence of asthma and respiratory symptoms according to demographic variables.

Disease/Respiratory Symptom	Total N = 3237	Habitation		*p*-Value	Sex		*p*-Value	Age (years)			*p*-Value
		Town n = 2960	Village n = 275		Boy n = 1571	Girl n = 1664		6–9 n = 637	10–13 n = 1237	14–16 n = 563	
Chronic cough	306 (9.5)	285 (9.6)	21 (7.6)	0.3	159 (10.1)	147 (8.8)	0.2	130 (11.1)	141 (8.5)	35 (8.6)	0.04
Wheezing in the last 12 months	298 (9.2)	280 (9.5)	18 (6.5)	0.1	165 (10.5)	133 (8.0)	0.01	130 (11.1)	136 (8.2)	32 (7.9)	0.02
Waking up at night due to wheezing	186 (5.8)	175 (5.9)	11 (4.0)	0.2	102 (6.5)	84 (5.0)	0.07	87 (7.5)	84 (5.0)	15 (3.7)	0.004
Dyspnea in the last 12 months	153 (4.7)	143 (4.8)	10 (3.6)	0.4	90 (5.7)	63 (3.8)	0.009	64 (5.5)	72 (4.3)	17 (4.2)	0.3
Asthma	298 (9.2)	270 (9.1)	28 (10.2)	0.6	180 (11.4)	118 (7.1)	<0.001	113 (9.7)	145 (8.7)	40 (9.8)	0.6

Data presented as numbers and percentages, n (%); *p*-value—the result of the chi-square test.

**Table 2:** The presence of pets in relation to the three scenarios.

Pets	Scenario 1	Pets	Scenario 2	Pets	Scenario 3
current	1796 (55.7)	current	1796 (55.7)	ever-present	1996 (62.0)
present in the past	202 (6.3)	no	1426 (44.3)	no	1224 (38.0)
no	1224 (38.0)				

Data presented as numbers and percentages, n (%).

**Table 3:** Presence of animals according to sex and age.

Variable	Pets			*p*-Value
	Current	Present in the Past	No	
Gender				
boy	820 (52.3)	93 (5.9)	656 (41.8)	<0.001
girl	976 (59.0)	109 (6.6)	568 (34.3)	
Age (years)				
6–9	594 (51.1)	55 (4.7)	514 (44.2)	<0.001
10–13	968 (58.4)	122 (7.4)	566 (34.2)	
14–16	234 (58.0)	25 (6.2)	144 (35.7)	

Data presented as numbers and percentages, n (%); *p*-value—the result of the chi-square test.

**Table 4:** Prevalence of asthma and respiratory symptoms according to the adopted scenario.

Disease/Respiratory Symptom	Scenario 1 Pets			*p*-Value	Scenario 2 Pets		*p*-Value	Scenario 3 Pets		*p*-Value
	Current	Present, in the Past	No		Current	No		Ever Present	No	
Chronic cough	170 (9.5)	17 (8.4)	118 (9.7)	0.8	170 (9.5)	135 (9.5)	1.0	187 (9.4)	118 (9.7)	0.7
Wheezing in the last 12 months	145 (8.1)	18 (8.9)	134 (10.9)	0.03	145 (8.1)	152 (10.7)	0.01	163 (8.2)	134 (11.0)	0.008
Waking up at night due to wheezing	90 (5.0)	9 (4.5)	87 (7.1)	0.04	90 (5.0)	96 (6.7)	0.04	99 (5.0)	87 (7.1)	0.01
Dyspnea in the last 12 months	80 (4.5)	9 (4.5)	64 (5.2)	0.6	80 (4.5)	73 (5.1)	0.4	89 (4.5)	64 (5.2)	0.3
Asthma	141 (7.9)	27 (13.4)	129 (10.5)	0.005	141 (7.9)	156 (10.9)	0.003	168 (8.4)	129 (10.5)	0.04

Data presented as numbers and percentages, n (%); *p*-value—the result of the chi-square test.

**Table 5:** Adjusted by sex and age odds ratios (OR) and 95% confidence intervals (95%CI) related to different scenarios.

Pets	Disease/Respiratory Symptom OR (95%CI) Adjusted by Sex and Age; *p*-Value									
	Chronic Cough OR (95%CI)	*p*-Value *	Wheeze in the Last 12 Months OR (95%CI)	*p*-Value *	Waking Up at Night due to Wheezing OR (95%CI)	*p*-Value *	Dyspnea in the Last 12 Months OR (95%CI)	*p*-Value *	Asthma OR (95%CI)	*p*-Value *
Scenario 1										
current/no ^Ref^ present, in the past/no ^Ref^	1.0 (0.8–1.3) 0.9 (0.5–1.5)	0.9 0.5	0.7 (0.6–0.9) 0.8 (0.5–1.4)	**0.02** 0.5	0.7 (0.5–0.9); 0.6 (0.3–1.3);	**0.04** 0.2	0.9 (0.6–1.2) 0.9 (0.4–1.8)	0.5 0.7	0.7 (0.5–0.9) 1.3 (0.8–2.1)	**0.02** 0.1
Scenario 2										
current/no ^Ref^	1.0 (0.8–1.3)	0.8	0.7 (0.6–0.9)	**0.02**	0.7 (0.5–1.0);	0.08	0.9 (0.6–1.2)	0.5	0.7 (0.6–0.9)	**0.007**
Scenario 3										
ever-present/no ^Ref^	1.0 (0.8–1.3)	0.9	0.7 (0.6–0.9)	**0.02**	0.7 (0.5–0.9);	**0.03**	0.9 (0.6–1.2)	0.5	0.8 (0.6–1.0)	0.09

Statistically significant *p*-values are indicated in bold. Ref—Reference Group. * Regression model adjusted for age and sex.

## Data Availability

Data are available on request due to restrictions, e.g., privacy or ethical. The data are not publicly available due to the survey protocol, which assured respondents of anonymity and that the data would not be shared with anyone not associated with the research team.
